# Supplementary material for: A Combination of In Silico ADMET Prediction, In Vivo Toxicity Evaluation, and Potential Mechanism Exploration of Brucine and Brucine N-oxide—A Comparative Study
Source: Molecules. 2023 Jan 31;28(3):1341. doi: 10.3390/molecules28031341 (PMC9919335; doi:10.3390/molecules28031341)
Supplement: Supplementary file 1 [file molecules-28-01341-s001.zip › molecules-2101074-supplementary.pdf]

1. Materials and Methods

Laboratory animals

Zebrafish (TU strain) were kept in an automatic circulation tank system and hold to the cycles of 14 h light and 10 h dark. The temperature of the sea water was retained at 28 ± 1.0°C with the pH at 7.0 ± 0.5. The embryos used for all the testing were gained from spawning adult zebrafish which kept in the breeding tanks. All of the experiments performed in this study were approved by the Committee on the Ethics of Animal Experiments of the Institute of Medicinal Biotechnology, Chinese Academy of Medical Sciences (Beijing, China, IMBF20060302).

Structures of brucine and brucine N-oxide

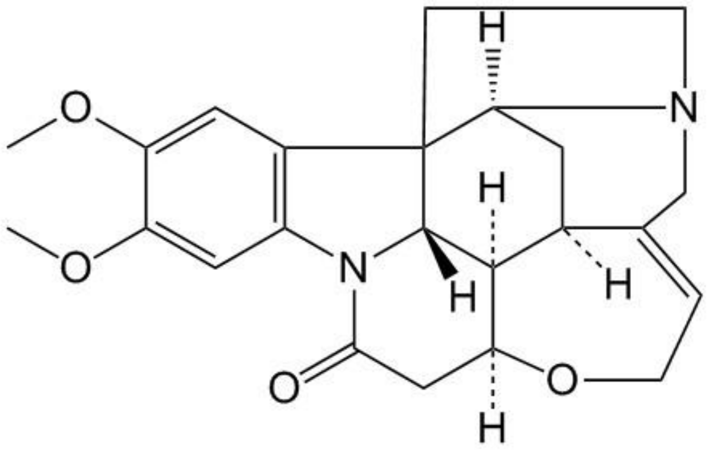

Figure S1. Structure of brucine.

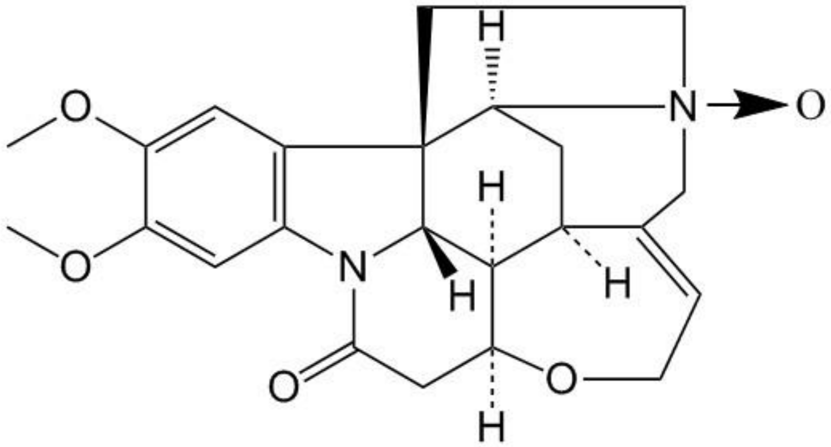

Figure S2. Structure of brucine N-oxide.

2. Results and discussions

Comments on the value of ADMET-related properties

Table S1. Comments on the value of physicochemical, medicinal chemical, and ADMET properties.

Property	Comment
Physicochemical Property	
TPSA	Topological Polar Surface Area. Optimal:0~140
LogS (Solubility)	Log of the aqueous solubility. Optimal: -4~0.5 log mol/L
LogD7.4(Distribution Coefficient D)	logP at physiological pH 7.4. Optimal: 1~3
LogP (Distribution Coefficient P)	Log of the octanol/water partition coefficient. Optimal: 0~3
Medicinal Chemistry	
QED	■ A measure of drug-likeness based on the concept of desirability;<br>■ Attractive: > 0.67; unattractive: 0.49~0.67; too complex: < 0.34
SAscore	■ Synthetic accessibility score is designed to estimate ease of synthesis of drug-like molecules.<br>■ SAscore ^{3} 6, difficult to synthesize; SAscore <6, easy to synthesize
Absorption	
Papp (Caco-2 Permeability)	Optimal: higher than -5.15 Log unit
Pgp-inhibitor	■ Category 1: Inhibitor; Category 0: Non-inhibitor;<br>■ The output value is the probability of being Pgp-inhibitor
Pgp-substrate	■ Category 1: substrate; Category 0: Non-substrate;

	■ The output value is the probability of being Pgp-substrate
HIA (Human Intestinal Absorption)	■ Human Intestinal Absorption<br>■ Category 1: HIA+(HIA < 30%); Category 0: HIA-(HIA < 30%); The output value is the probability of being HIA+
Distribution	
PPB (Plasma Protein Binding)	■ Plasma Protein Binding<br>■ Optimal: < 90%. Drugs with high protein-bound may have a low therapeutic index.
VD (Volume Distribution)	■ Volume Distribution<br>■ Optimal: 0.04-20L/kg
BBB (Blood–Brain Barrier)	■ Blood-Brain Barrier Penetration<br>■ Category 1: BBB+; Category 0: BBB-; The output value is the probability of being BBB+
Metabolism	
P450 CYP1A2 inhibitor	■ Category 1: Inhibitor; Category 0: Non-inhibitor;<br>■ The output value is the probability of being inhibitor.
P450 CYP1A2 Substrate	■ Category 1: Substrate; Category 0: Non-substrate;<br>■ The output value is the probability of being substrate.
P450 CYP3A4 inhibitor	■ Category 1: Inhibitor; Category 0: Non-inhibitor;<br>■ The output value is the probability of being inhibitor.
P450 CYP3A4 substrate	■ Category 1: Substrate; Category 0: Non-substrate;<br>■ The output value is the probability of being substrate.
P450 CYP2C9 inhibitor	■ Category 1: Substrate; Category 0: Non-substrate;<br>■ The output value is the probability of being substrate.
P450 CYP2C9 substrate	■ Category 1: Substrate; Category 0: Non-substrate;<br>■ The output value is the probability of being substrate.
P450 CYP2C19 inhibitor	■ Category 1: Inhibitor; Category 0: Non-inhibitor;<br>■ The output value is the probability of being

	inhibitor.
P450 CYP2C19 substrate	■ Category 1: Substrate; Category 0: Non-substrate;<br>■ The output value is the probability of being substrate.
P450 CYP2D6 inhibitor	■ Category 1: Inhibitor; Category 0: Non-inhibitor;<br>■ The output value is the probability of being inhibitor.
P450 CYP2D6 substrate	■ Category 1: Substrate; Category 0: Non-substrate;<br>■ The output value is the probability of being substrate.
Elimination	
T 1/2 (Half Life Time)	■ Category 1: long half-life ; Category 0: short half-life;<br>■ long half-life: >3h; short half-life: <3h<br>■ The output value is the probability of having long half-life.
CL (Clearance Rate)	■ Clearance<br>■ High: >15 mL/min/kg; moderate: 5-15 mL/min/kg; low: <5 mL/min/kg
Toxicity	
hERG (hERG Blockers)	■ Category 1: active; Category 0: inactive;<br>■ The output value is the probability of being active.
H-HT (Human Hepatotoxicity)	■ Category 1: active; Category 0: inactive;<br>■ The output value is the probability of being active.
AMES (Ames Mutagenicity)	■ Category 1: active; Category 0: inactive;<br>■ The output value is the probability of being active.
SkinSen (Skin sensitization)	■ Category 1: Sensitizer; Category 0: Non-sensitizer;<br>■ The output value is the probability of being sensitizer.
DILI (Drug Induced Liver Injury)	■ Drug Induced Liver Injury.<br>■ Category 1: drugs with a high risk of DILI; Category<br>0: drugs with no risk of DILI. The output value is the probability of being toxic.
